# A Positive Feedback Loop of Hippo- and c-Jun-Amino-Terminal Kinase Signaling Pathways Regulates Amyloid-Beta-Mediated Neurodegeneration

**DOI:** 10.3389/fcell.2020.00117

**Published:** 2020-03-13

**Authors:** Madison Irwin, Meghana Tare, Aditi Singh, Oorvashi Roy Puli, Neha Gogia, Matthew Riccetti, Prajakta Deshpande, Madhuri Kango-Singh, Amit Singh

**Affiliations:** ^1^Department of Biology, University of Dayton, Dayton, OH, United States; ^2^Premedical Program, University of Dayton, Dayton, OH, United States; ^3^Center for Tissue Regeneration and Engineering at Dayton (TREND), University of Dayton, Dayton, OH, United States; ^4^The Integrative Science and Engineering Center, University of Dayton, Dayton, OH, United States; ^5^Center for Genomic Advocacy (TCGA), Indiana State University, Terre Haute, IN, United States

**Keywords:** neurodegeneration, Alzheimer's disease, cell death, amyloid-beta 42, Hippo signaling, growth regulation, c-Jun-amino-terminal kinase (JNK) signaling, *Drosophila* eye

## Abstract

Alzheimer's disease (AD, OMIM: 104300) is an age-related disorder that affects millions of people. One of the underlying causes of AD is generation of hydrophobic amyloid-beta 42 (Aβ42) peptides that accumulate to form amyloid plaques. These plaques induce oxidative stress and aberrant signaling, which result in the death of neurons and other pathologies linked to neurodegeneration. We have developed a *Drosophila* eye model of AD by targeted misexpression of human Aβ42 in the differentiating retinal neurons, where an accumulation of Aβ42 triggers a characteristic neurodegenerative phenotype. In a forward deficiency screen to look for genetic modifiers, we identified a molecularly defined deficiency, which suppresses Aβ42-mediated neurodegeneration. This deficiency uncovers *hippo* (*hpo*) gene, a member of evolutionarily conserved Hippo signaling pathway that regulates growth. Activation of Hippo signaling causes cell death, whereas downregulation of Hippo signaling triggers cell proliferation. We found that Hippo signaling is activated in Aβ42-mediated neurodegeneration. Downregulation of Hippo signaling rescues the Aβ42-mediated neurodegeneration, whereas upregulation of Hippo signaling enhances the Aβ42-mediated neurodegeneration phenotypes. It is known that c-Jun-amino-terminal kinase (JNK) signaling pathway is upregulated in AD. We found that activation of JNK signaling enhances the Aβ42-mediated neurodegeneration, whereas downregulation of JNK signaling rescues the Aβ42-mediated neurodegeneration. We tested the nature of interactions between Hippo signaling and JNK signaling in Aβ42-mediated neurodegeneration using genetic epistasis approach. Our data suggest that Hippo signaling and JNK signaling, two independent signaling pathways, act synergistically upon accumulation of Aβ42 plaques to trigger cell death. Our studies demonstrate a novel role of Hippo signaling pathway in Aβ42-mediated neurodegeneration.

## Introduction

Alzheimer's disease (AD) is a progressive neurodegenerative disorder that affects the aging population and is predicted to continually increase in prevalence and incidence in the United States (Barnes and Yaffe, 2011). The hallmark of AD and other neurodegenerative diseases is loss of cognitive function due to neuronal death (Hardy, 2009; Hirth, 2010; O'Brien and Wong, 2010; Selkoe and Hardy, 2016). AD is characterized by accumulation of two types of protein aggregates in AD brains, *viz*., extracellular plaques of amyloid-beta (Aβ) peptides and intracellular tangles of hyper-phosphorylated and cleaved forms of tau, the microtubule-associated protein (MAP). Abnormal cleavage of the amyloid precursor protein (APP) results in 42 amino acid long polypeptides hereafter referred to as Aβ42 peptides (Crews and Masliah, 2010; O'Brien and Wong, 2010; Selkoe and Hardy, 2016). As per the amyloid hypothesis, the accumulation of the Aβ42 peptides into plaques initiates a pathological cascade eventually leading to neurodegeneration (Tare et al., 2011; Selkoe and Hardy, 2016; Yeates et al., 2019). Since this hypothesis was postulated, several signaling pathways and genetic modifiers have been implicated in Aβ42-mediated neurodegeneration (Moran et al., 2013; Steffensmeier et al., 2013; Cutler et al., 2015; Yeates et al., 2019).

Since the genetic machinery is highly conserved, many AD animal models including mouse, rodents, flies, fish, dogs, and non-human primates have been developed to discern mechanisms of neurodegeneration (Iijima-Ando and Iijima, 2010; Pandey and Nichols, 2011; Tare et al., 2011; Sabbagh et al., 2013). These animal models also allow testing for therapeutic targets (Pandey and Nichols, 2011; Sabbagh et al., 2013; Sarkar et al., 2016, 2018b; Deshpande et al., 2019). *Drosophila melanogaster*, the fruit fly, has served as a versatile model to study neurodegenerative diseases (Hirth, 2010; Prussing et al., 2013). The adult *Drosophila* eye arises from a monolayer epithelium, which is housed inside the larva and is referred to as the eye-antennal imaginal disc (Kumar, 2011; Singh et al., 2012; Tare et al., 2013). The adult eye is comprised of nearly 800 unit eyes or ommatidia (Ready et al., 1976; Kumar, 2011; Singh et al., 2012). After retinal differentiation, few undifferentiated cells undergo programmed cell death (PCD) during pupal development (Brachmann and Cagan, 2003). It is notable that PCD does not normally occur during early eye development; however, cell death may occur due to abnormal signaling (Mehlen et al., 2005; Singh et al., 2006; Tare et al., 2016). We have developed a *Drosophila* AD model by misexpressing high levels of human Aβ42 polypeptide in the differentiating photoreceptor neurons of the developing *Drosophila* eye. Misexpression of Aβ42 in the developing *Drosophila* eye results in progressive loss of photoreceptor neurons and aberrant morphology that mimics the neuropathology of atrophy and loss of neurons linked to AD (Tare et al., 2011; Sarkar et al., 2016).

Activation of the c-Jun-amino-terminal kinase (JNK) signaling pathway is implicated in Aβ42-mediated neurodegeneration (Tare et al., 2011; Sarkar et al., 2016). JNK signaling, which belongs to the mitogen-activated protein kinase (MAPK) superfamily, is a stress-activated protein kinase that triggers apoptosis upon activation (Adachi-Yamada and O'connor, 2004; Stronach, 2005; Dhanasekaran and Reddy, 2008). The JNK cascade is initiated by the binding of the ligand Eiger (Egr), the *Drosophila* homolog of the human tumor necrosis factor (TNF) to TNF receptors, named Wengen and Grindelwald in flies (Igaki et al., 2002; Kanda et al., 2002; Moreno et al., 2002). Upon receptor activation, the signal is transmitted by *hemipterous* (*hep*), the *Drosophila* JNKK that phosphorylates *basket* (*bsk*), the *Drosophila* JNK (Glise et al., 1995; Sluss et al., 1996; Holland et al., 1997). Bsk phosphorylates and activates *Drosophila* Jun-related antigen (Jra or dJun). The transcription factor Jun translocates to the nucleus to induce target genes of the JNK pathway (Sluss et al., 1996; Kockel et al., 2001). A key transcriptional target of JNK signaling is *puckered* (*puc*), which is a dual-specificity phosphatase that negatively regulates *bsk* and thereby forms a negative feedback loop (Martin-Blanco et al., 1998; Adachi-Yamada, 2002; Stronach, 2005). When activated, JNK signaling triggers cell death by phosphorylation of *reaper* (*rpr)* and *head involution defective* (*hid*), as well as caspase-independent mechanisms (Martin-Blanco et al., 1998; Stronach, 2005; Singh et al., 2006; Igaki, 2009).

Interestingly, a key interactor of the JNK is the Hippo pathway, which is a conserved signaling pathway primarily involved in the regulation of organ size (Kango-Singh and Singh, 2009; Pan, 2010; Halder and Johnson, 2011; Staley and Irvine, 2012). The Hippo and JNK pathways interact in several contexts, for example, in the regulation of growth, cell survival, and regeneration (Grusche et al., 2011; Sun and Irvine, 2011). The Hippo pathway is comprised of several upstream regulators that relay the signal through a core kinase cascade that ultimately controls the activation of Yorkie (Yki), the effector of the Hippo pathway (Huang et al., 2005). Yki acts as a transcriptional coactivator and requires Scalloped (Sd), a TEAD/TEF family transcription factor to induce the expression of Hippo pathway target genes (Wu et al., 2008; Zhang et al., 2008; Ren et al., 2010). The core kinase cassette is comprised of Hippo (Hpo) and Warts (Wts), two serine-threonine protein kinases of the mammalian Ste-20 and nuclear Dbf-2-related (NDR) kinase family, respectively. Hpo phosphorylates and complexes with the WW-domain containing adaptor protein Salvador (Sav). The Hpo–Sav complex interacts with the downstream kinase Wts and its binding partner Mob-as-tumor-suppressor (Mats). Following Hpo-mediated phosphorylation, Wts undergoes autophosphorylation and in turn phosphorylates Yki (Justice et al., 1995; Kango-Singh et al., 2002; Tapon et al., 2002; Harvey et al., 2003; Jia et al., 2003; Pantalacci et al., 2003; Udan et al., 2003; Wu et al., 2003; Huang et al., 2005; Lai et al., 2005; Wei et al., 2007; Kango-Singh and Singh, 2009). Overall, activation of the Hippo pathway sequesters Yki in the cytoplasm and results in induction of cell death and decreased organ size (Harvey et al., 2003; Pantalacci et al., 2003; Udan et al., 2003; Wu et al., 2003; Wei et al., 2007; Verghese et al., 2012). In contrast, inactivation or downregulation of Hippo pathway allows Yki to translocate to the nucleus, bind Sd, and regulate expression of target genes. These target genes include *Myc* and *bantam*, the two promoters of growth, and *Diap1*, a *Drosophila* inhibitor of apoptosis protein 1 (Nolo et al., 2006; Thompson and Cohen, 2006; Wu et al., 2008; Zhang et al., 2008; Peng et al., 2009; Neto-Silva et al., 2010; Oh and Irvine, 2011). Other phosphorylation-independent mechanisms of Yki regulation are also known that mainly involve physical association of Yki with Hippo signaling components, which prevents its nuclear localization (Oh and Irvine, 2008, 2009, 2010; Zhang et al., 2008). While Hippo signaling plays a role in several diseases like cancer, polycystic kidney disease, and heart disease, its role in neurodegenerative diseases such as AD remains poorly understood.

In a genetic modifier screen, we identified a deficiency, *Df(2R)BSC782/*+, which rescued the Aβ42-mediated neurodegeneration phenotype. This deficiency uncovers 10 genes including the *hpo* gene. Further testing with the candidate genes uncovered by *Df(2R)BSC782* revealed *hpo* as the causal genetic modifier of the neurodegeneration phenotype of Aβ42 overexpression. This suggested that *hpo* and other components of the Hippo signaling pathway may impact the Aβ42-mediated neurodegeneration phenotype. Here we report that the Hippo pathway affects Aβ42-mediated neurodegeneration phenotypes as hyperactivation of Hippo signaling leads to enhancement of Aβ42 toxicity, whereas downregulation of Hippo signaling rescues Aβ42-mediated neurodegeneration phenotype. Previously, we had reported that Aβ42 induced neuronal apoptosis *via* activation of a JNK–caspase-dependent pathway. Recently, JNK and Hippo pathway were shown to interact in several contexts, which prompted us to study if JNK–Hippo interactions affected the Aβ42-mediated neurodegeneration phenotype. Here we report that misexpression of Aβ42 induces JNK signaling, which in turn, induces Hippo signaling by blocking Yki activation. Activation of Hippo signaling in Aβ42-mediated neurodegeneration activates *puc*-lacZ, a reporter of JNK signaling. Here we present evidences to support a role for a positive feedback loop between JNK and Hippo signaling pathways that promotes Aβ42-mediated neurodegeneration in the *Drosophila* eye.

## Materials and Methods

### Fly Stocks

All fly stocks used in this study are listed at FlyBase (http://flybase.bio.indiana.edu). Fly stocks used in this study were: GMR-Gal4 (Moses and Rubin, 1991), UAS-Aβ42 (Tare et al., 2011; Sarkar et al., 2018b), UAS-*hpo* (Udan et al., 2003), UAS-*hpo*^*RNAi*^ (Pantalacci et al., 2003), UAS-*wts*^13F^ (Kwon et al., 2015), UAS-*wts*^*RNAi*^ (Trip Line), UAS-*yki* (Oh and Irvine, 2009), UAS-*yki*^RNAi(N+C)^ (Zhang et al., 2008), *diap1-4.3*-green fluorescent protein (GFP) (Ren et al., 2010), *hid 5*′*-*GFP (Tanaka-Matakatsu et al., 2009), *ex*^697^*-lacZ* (Boedigheimer et al., 1997), UAS-*puc, puc*^*E*69^ (Martin-Blanco et al., 1998), UAS-*jun*^*aspv*7^ (Treier et al., 1995), UAS-*hep*^Act^ (Glise et al., 1995), UAS-*b*s*k*^DN^ (Adachi-Yamada et al., 1999). For the genetic screen, we used molecularly defined deficiencies. We identified *Df(2R)BSC782/*+, a deficiency, which is located on the right arm of the second chromosome, and uncovers β*Tub56D, par-1, CG16926, CG7744, CG15120, mei-W68, oseg6, TBCB, rep*, and *hpo* genes (listed in Flybase). For wild-type control, we used the Canton-S stock of *D. melanogaster* in this study. Fly stocks were maintained at 25°C on the regular cornmeal, yeast, molasses food medium.

### Genetic Crosses

We employed a Gal4/UAS system for targeted misexpression studies (Brand and Perrimon, 1993). All Gal4/UAS crosses were maintained at 18, 25, and 29°C, unless specified, to sample different induction levels (Singh and Choi, 2003; Singh et al., 2005). The GMR-Gal4 driver used in this study targets misexpression of transgenes in the differentiating retinal precursor cells of the developing eye imaginal disc and pupal retina (Moses and Rubin, 1991). Misexpression of Aβ42 in the differentiating retina (GMR-Gal4 > UAS-Aβ42, referred to as GMR> Aβ42 throughout the text) exhibits a stronger neurodegenerative phenotype at 29°C (Tare et al., 2011; Sarkar et al., 2018b). For all other genetic interaction studies involving the JNK and Hippo pathway, UAS lines that upregulate or downregulate pathway components were tested using appropriate transgenes by crossing to the GMR> Aβ42 flies though appropriate genetic crosses.

### Immunohistochemistry

Eye-antennal imaginal discs were dissected from the wandering third-instar larvae in 1X phosphate buffered saline (PBS), fixed in 4% paraformaldehyde in PBS for 20 min, and washed in PBS. We stained the tissue with a combination of antibodies following a previously published protocol (Singh et al., 2002; Sarkar et al., 2018a). The primary antibodies used were rat anti-Embryonic Lethal Abnormal Vision (ELAV) (1:50; Developmental Studies Hybridoma Bank, DSHB), mouse anti-discs large (Dlg) (1:100; DSHB), rabbit anti-Dlg (1:200; a gift from Dr. K. Cho), mouse anti-6E10 (1:100), rabbit anti-β-galactosidase (1:200; Cappel), mouse anti-22C10 (1:100; DSHB), and mouse anti-Chaoptin (MAb24B10) (1:100; DSHB) (Zipursky et al., 1984). Secondary antibodies (Jackson Laboratory) used were goat anti-rat IgG conjugated with Cy5 (1:250), donkey anti-rabbit IgG conjugated with fluorescein isothiocyanate (FITC) (1:200), donkey anti-mouse IgG conjugated with FITC (1:200), and donkey anti-mouse IgG conjugated with Cy3 (1:250). We mounted the tissues in Vectashield (Vector Laboratories). The immunofluorescent images were captured by laser scanning confocal microscopy (Singh and Gopinathan, 1998). We took the images at 20× magnification unless stated otherwise. We analyzed and prepared the final figures with images using Adobe Photoshop CS6 software.

### Detection of Cell Death

We performed terminal deoxynucleotidyl transferase dUTP nick end labeling (TUNEL) assays using a cell death detection kit from Roche Diagnostics. TUNEL assays allow identification of the cells undergoing cell death where the cleavage of double- and single-stranded DNA is labeled by a fluorescent tag (TMR Red) (White et al., 1994; Mccall and Peterson, 2004). The fluorescently labeled nucleotides are added to 3′ OH ends in a template-independent manner by terminal deoxynucleotidyl transferase (TdT). The fluorescent-tagged fragmented DNA within a dying cell can be detected by fluorescence microscopy. After secondary antibody staining, eye antennal discs were blocked in 10% normal donkey serum in PBS with 0.2% Triton X-100 (PBT) and processed for TUNEL labeling (Singh et al., 2006). For each genotype, we counted TUNEL-positive nuclei from five sets of eye imaginal discs to determine dying cell population. We used these cell counts for statistical analysis using Microsoft Excel 2013. We calculated the *p*-values using Student two-tailed *t*-test, and the error bars represent standard deviation from mean.

### Adult Eye Imaging

We prepared the adult flies for imaging by freezing at −20°C for ~2 h followed by mounting the fly on a dissection needle. The needle was secured with mounting putty to suspend the fly horizontally over a glass slide. We took adult eye images on the Axiomager.Z1 Zeiss Apotome and obtained the Z-stacks (Oros et al., 2010; Wittkorn et al., 2015; Singh et al., 2019). Final images are projections of Z-stacks using the extended depth of focus function of the Axiovision software 4.6.3.

### Western Blot

Protein samples were prepared from (*n* = ~50) adult eyes from Canton-S (wild-type), GMR> Aβ42, GMR> Aβ42+*hpo* following standardized protocols (Gogia et al., 2017). The samples were loaded in the following sequence: Lane 1-Molecular weight marker (BIORAD Precision Plus Protein Kaleidoscope Prestained Catalog Number #1610375), Lane 2-Wild-type (Canton-S), Lane 3-GMR> Aβ42, Lane 4-GMR> Aβ42+*hpo* (gain-of-function), Lane 5-GMR> Aβ42+*hpo*^*RNAi*^ (loss-of-function). We incubated the blots with the Phospho SAPK/JNK (81E11) (1:3,000, Cell Signaling) and after appropriate washes incubated with horseradish peroxidase conjugated goat anti–rabbit IgG (1:5,000) secondary antibody. Signal was detected using super signal chemiluminescence substrate (Pierce). We captured the images using the BioSpectrum® 500 Imaging System and analyzed the blot images and band intensity. We used Microsoft Excel 2017 software for statistical analysis. We calculated the *p*-values using the Student two-tailed *t*-test. The error bars represent standard deviation from means.

## Results

### Genetic Modifier of Amyloid-Beta 42-Mediated Neurodegeneration

**Figure 1:**
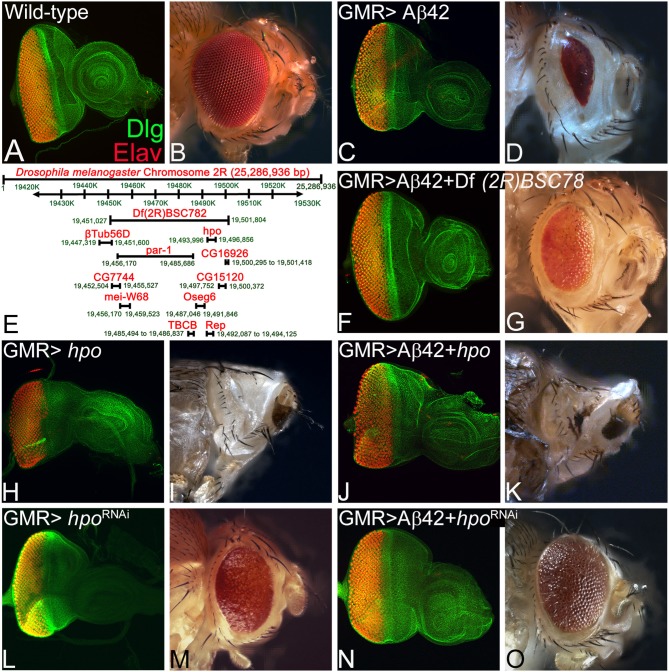
*hippo* is a genetic modifier of amyloid-beta 42 (Aβ42)-mediated neurodegeneration in the *Drosophila* eye. Panels show images of eye imaginal discs stained for the proneural marker embryonic lethal abnormal vision (ELAV; red) and a membrane-specific marker discs large (Dlg; green), and the resulting eye phenotype in the adult from **(A,B)** wild-type and **(C,D)** glass multiple repeat (GMR)> Aβ42. **(D)** Note that the GMR> Aβ42 adult eyes are highly reduced and have glazed morphology with black necrotic spots. **(E)** A map showing the deficiency BSC782 identified in the forward genetic screen and position of *hpo* and other genes within this deficiency is depicted. **(F–O)** Panels show the eye disc stained with Dlg (green) and ELAV (red) and accompanying adult eye phenotypes from **(F,G)** GMR> Aβ42 + *Df(2R)BSC782/*+, **(H,I)** GMR> *hpo*, **(J,K)** GMR> Aβ42 + *hpo*, **(L,M)** GMR> *hpo*^RNAi^, and **(N,O)** GMR> Aβ42 + *hpo*^RNAi^. Note that downregulation of Hippo signaling **(N,O)** GMR> Aβ42 + *hpo*^RNAi^ significantly rescues the GMR> Aβ42 neurodegenerative phenotype, whereas activation of Hippo signaling **(J,K)** GMR> Aβ42 + *hpo* enhances the GMR> Aβ42 neurodegenerative phenotype. The orientation of all imaginal discs is identical with posterior to the left and dorsal up. Magnification of the eye disc or adult eye images is the same across all panels.

The wild-type eye imaginal disc (Figure 1A) develops into the adult compound eye (Figure 1B). The eye-antennal imaginal discs were stained with a membrane-specific marker Dlg (green) and pan neural marker ELAV (red), which marks the nuclei of the photoreceptor neurons (Figure 1A). Targeted misexpression of human Aβ42 in the differentiating photoreceptor neuron of the developing eye imaginal disc using GMR-Gal4 driver (GMR> Aβ42) results in the loss of photoreceptor neurons on the posterior margin of the eye imaginal disc (Figure 1C). The neurodegeneration phenotype worsens with time, which results in a highly reduced adult eye with glazed appearance [*n* = 112, all of them (112/112, 100%) showed highly reduced adult eye phenotype, Figure 1D] (Tare et al., 2011).

We performed a forward genetic screen using molecularly defined deficiencies to find genetic modifiers of Aβ42 neurodegeneration phenotype. In this screen, we identified a deficiency, *Df(2R)BSC782*, which in transheterozygous combination (*Df(2R)BSC782/*+) rescues the GMR> Aβ42-mediated neurodegeneration phenotype both in the eye imaginal disc (Figure 1F) and the adult eye (*n* = 117, 89/117, 76% of the adult eye showed rescue phenotype; Figure 1G). Interestingly, *Df(2R)BSC782* deficiency is located on the right arm of the second chromosome and uncovers 10 genes including *hippo* (*hpo*), a member of highly conserved Hippo growth regulatory pathway (Figure 1E). We individually tested genes uncovered by *Df(2R)BSC782* (Figure 1K) using gain-of-function and loss-of-function approaches to identify which gene(s) functions as the genetic modifier(s) of Aβ42 (GMR> Aβ42)-mediated neurodegeneration in the *Drosophila* eye. Misexpression of *hpo* (GMR>*hpo)* results in the loss of photoreceptor neurons on the posterior margin of the eye imaginal disc (Figure 1H), resulting in a highly reduced or “No-eye” phenotype in the adult fly (n = 119, 108/119, 91% showed reduced or “No-eye” phenotype; Figure 1I). As compared to GMR> Aβ42 (Figures 1C,D), misexpression of *hpo* along with Aβ42 (GMR> Aβ42+ *hpo*) enhances neuronal loss and results in a stronger neurodegeneration phenotype in the eye imaginal disc (Figure 1J) and the adult eye (*n* = 136, 136/136, 100% showed strong neurodegenerative phenotype; Figure 1K). The GMR> Aβ42+ *hpo* adults, which showed strong pupal lethality, were dissected out from their pupal cases as they failed to close. To further validate the role of Hippo signaling, we downregulated *hpo* gene function by misexpressing *hpo*^RNAi^ (GMR> *hpo*^RNAi^) that results in mild overgrowth both in the eye disc (Figure 1L) and in the adult flies (*n* = 98, 61/98, 62%; Figure 1M). Coexpression of *hpo*^RNAi^ with Aβ42 (GMR> Aβ42+ *hpo*^RNAi^) shows a strong rescue of Aβ42-mediated neurodegeneration both in the eye imaginal disc (Figure 1N) and the adult eye (*n* = 107, 71/107, 66%; Figure 1O). The adults of GMR> Aβ42+ *hpo*^RNAi^ show a dramatic rescue to near wild-type adult eye and significantly reduced the pupal lethality as compared to GMR> Aβ42 or GMR> Aβ42+ *hpo*. These results validate our findings from deficiency screen that *hpo* is a genetic modifier of Aβ42-mediated neurodegeneration in the *Drosophila* eye. In order to understand the mechanism of Aβ42-mediated neurodegeneration, it is important to understand the impact of Hippo signaling on the Aβ42 and its downstream effects.

### Modulation of Hippo Activity Does Not Affect Amyloid-Beta 42 Plaque Accumulation

**Figure 2:**
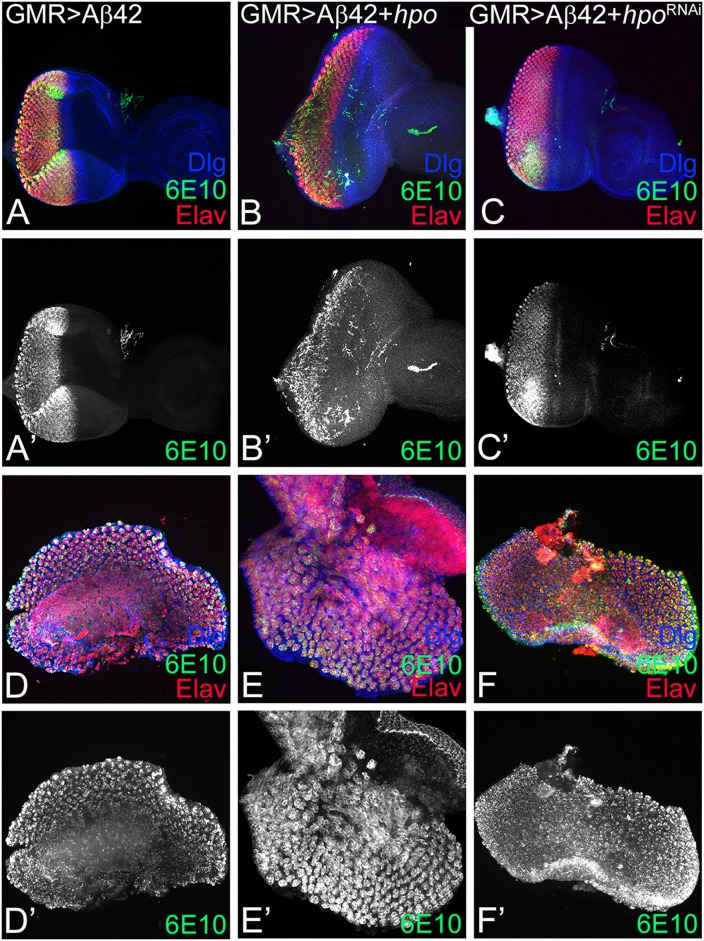
*hpo* does not affect the amyloid-beta 42 (Aβ42) accumulation. Panels show eye imaginal discs and pupal retinae stained with the proneural marker embryonic lethal abnormal vision (ELAV; shown in red); 6E10, an anti-Aβ42 antibody (green or gray), and the membrane-specific marker discs large (Dlg; blue). **(A–C)** shows confocal images of eye discs for all markers (Dlg, 6E10, ELAV), whereas the 6E10 expression alone (gray) is shown in panels **(A'–C')**. Eye discs **(A–C)** and pupal retinae **(D–F)** of the following genotypes were compared: **(A,A',D,D')** glass multiple repeat (GMR)> Aβ42, **(B,B',E,E')** GMR> Aβ42 + *hpo*, and **(C,C',F,F')** GMR> Aβ42 + *hpo*^RNAi^. Note that activation (GMR> Aβ42 + *hpo*) or downregulation (GMR> Aβ42 + *hpo*^RNAi^) of Hippo signaling in GMR> Aβ42 background does not affect the accumulation of Aβ42 plaques.

Since loss-of-function of *hpo* can rescue Aβ42-mediated neurodegeneration, we therefore tested the effects of modulation of Hippo signaling on Aβ42 accumulation. We used the 6E10 antibody that specifically detects the Aβ42 polypeptide. We observed robust Aβ42 accumulation in the eye imaginal discs from GMR> Aβ42 (Figures 2A,A′), GMR> Aβ42+ *hpo* (Figures 2B,B′), or GMR> Aβ42+ *hpo*^*RNAi*^ (Figures 2C,C′), suggesting that upregulation or downregulation of Hpo does not directly affect the levels of Aβ42 in the eye disc. It is noteworthy that changes in Hpo levels have a significant effect on GMR> Aβ42 (Figures 2A,A′) phenotypes where GMR> Aβ42+ *hpo* (Figures 2B,B′) enhances neurodegeneration, and GMR> Aβ42+ *hpo*^*RNAi*^ (Figures 2C, C′) show a significant rescue of the Aβ42-mediated neurodegenerative phenotype.

GMR enhancer drives expression in the differentiating retinal neurons, which initiates in early third instar of eye development, and continues in pupal retina. Since accumulation of Aβ42 exhibits a progressive neurodegenerative phenotype, we analyzed the effect of modulation of *hpo* levels at a later time window of pupal development. Interestingly, Aβ42 accumulation is not affected in the pupal retina of GMR> Aβ42 (Figures 2D,D′), GMR> Aβ42+ *hpo* (Figures 2E,E′), or GMR> Aβ42+ *hpo*^*RNAi*^ (Figures 2F,F′). This suggests that changes in the *hpo* activity do not affect Aβ42 expression or levels during larval or pupal stages. Thus, *hpo* may affect the Aβ42 phenotypes by acting downstream of Aβ42 accumulation likely by modifying downstream signals.

### Hippo Signaling Is Activated in Amyloid-Beta 42-Mediated Neurodegeneration

**Figure 3:**
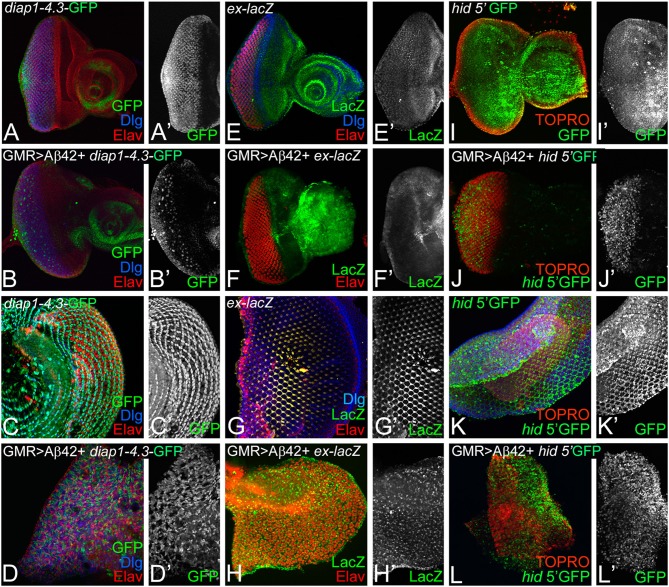
Accumulation of amyloid-beta 42 (Aβ42) activates Hippo signaling. Expression levels of Hippo pathway reporters were tested in eye discs and pupal retinae. Expression of *diap1 4.3*-green fluorescent protein (GFP) (green, gray) is shown for eye discs and pupal retinae from **(A,A',C,C')** wild-type, **(B,B',D,D')** glass multiple repeat (GMR)> Aβ42, respectively. *diap1-4.3-GFP* expression is shown in a split channel in gray in **(C',D')** panels. Changes in *ex-lacZ* (green, gray) levels is shown in eye discs and pupal retinae from **(E,E',F,F')** wild type, **(G,G',H,H')** GMR> Aβ42, respectively. *ex-lacZ* expression is shown in a split channel in gray in **(E'–H')**. **(I–L)** shows the expression of *hid*-5' GFP (green), a reporter for cell death in wild-type **(I,I')** eye imaginal disc and **(K,K')** pupal retina and GMR> Aβ42 **(J,J')** eye imaginal disc and the **(L,L')** pupal retina. Gray panels **(I'–L')** show *hid*-5' GFP expression in indicated genotypes. In **(I–L)**, nuclei are marked by the nuclear dye TOPRO (red).

One possibility is that Aβ42 accumulation may cause neurodegeneration by affecting Hippo pathway activity. Therefore, we investigated if expression of Hippo pathway reporters is affected in GMR> Aβ42 background (Figure 3). *diap1- 4.3*-GFP and *ex-*lacZ serve as functional readouts of the Hippo signaling pathway (Hamaratoglu et al., 2006; Kango-Singh and Singh, 2009; Ren et al., 2010). *diap1-4.3*-GFP is a Hippo response element mapped to the regulatory regions of *diap1* gene, which reports *diap 1* endogenous gene activity in response to Hippo signaling by changes in the expression of a GFP reporter (Ren et al., 2010). In the wild type, *diap1* is expressed uniformly in the eye region of the imaginal disc (Figures 3A,A′) and ubiquitously in the pupal retina (Figures 3C,C′). Loss-of-function of *hpo* results in cell proliferation along with upregulation of *diap1* reporter in the eye disc (Ren et al., 2010). Misexpression of Aβ42 (GMR> Aβ42+*diap1-*4.3-GFP) results in a strong suppression of the *diap1-4.3*-GFP reporter expression in the GMR>Aβ42 eye imaginal disc (Figures 3B,B′) and pupal retina (Figures 3D,D′). Downregulation of *diap1-4.3*-GFP reporter suggests that Hippo signaling is activated in the GMR>Aβ42 background. Similarly, another reporter of Hippo signaling activity, *ex*-lacZ, is expressed ubiquitously in the wild-type eye imaginal disc (Figures 3E,E′) and in the pupal retina (Figures 3G,G′). However, *ex*-lacZ expression is downregulated in GMR> Aβ42 background both in the eye imaginal disc (Figures 3F,F′) and in the pupal retina (Figures 3H,H′). Activation of Hippo pathway is known to upregulate *hid* expression (Udan et al., 2003). Therefore, we tested the *hid5*′-GFP reporter. In comparison to the wild-type eye imaginal disc (Figures 3I,I′), *hid5*′-GFP is robustly induced in GMR> Aβ42 eye imaginal disc (Figures 3J,J′). Similarly, *hid5*′-GFP was strongly upregulated in the pupal retina of GMR> Aβ42 (Figures 3L,L′) as compared to the wild-type pupal retina (Figures 3K,K′). Thus, misexpression of Aβ42 (GMR> Aβ42) activates Hippo signaling that results in induction of cell death.

### Hippo Signaling Levels Affect Amyloid-Beta 42-Mediated Neuronal Cell Death

**Figure 4:**
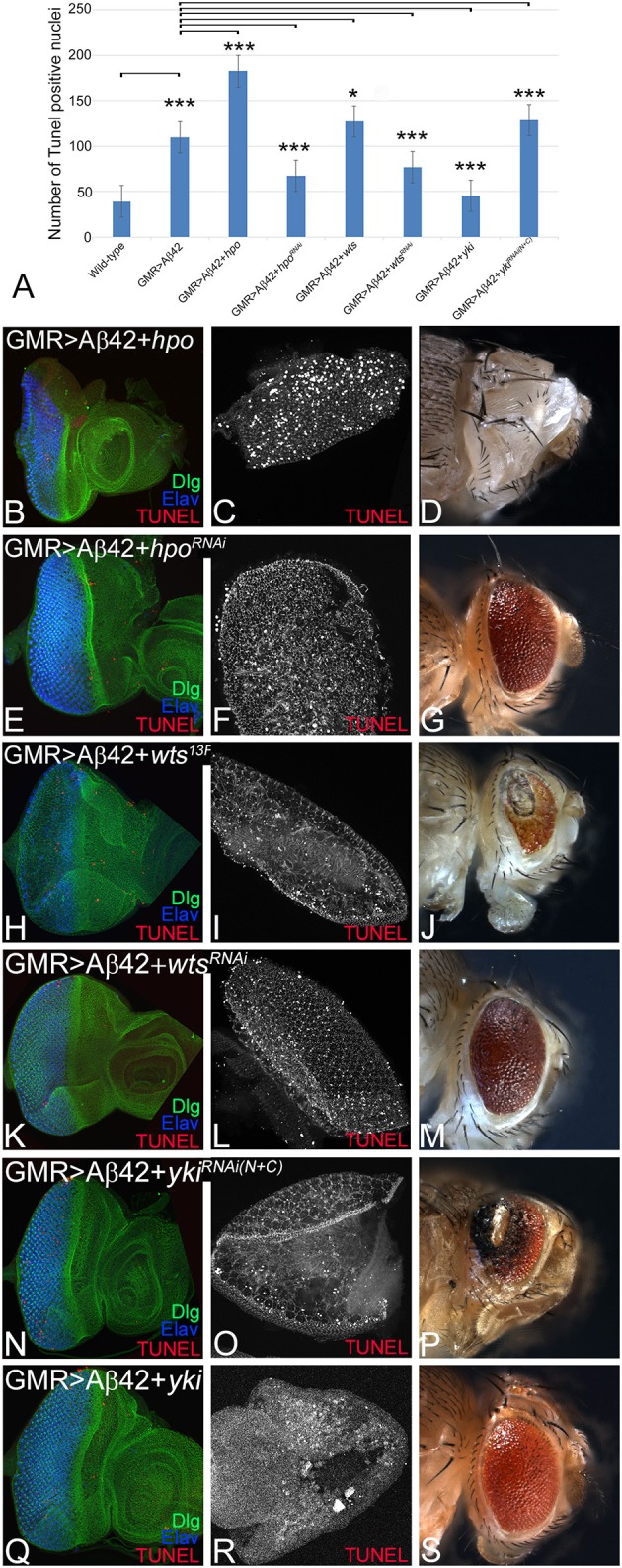
Hippo activation triggers cell death in glass multiple repeat (GMR)> amyloid-beta 42 (Aβ42) background. For each genotype, we counted terminal deoxynucleotidyl transferase dUTP nick end labeling (TUNEL)-positive nuclei from five (*n* = 5) eye imaginal discs to determine dying cell population. **(A)** Quantification of TUNEL-positive nuclei in indicated genotypes is shown (*n* = 5, *p* ≤ 0.05). The *p*-values obtained from Student's *t*-test between wild-type and GMR> Aβ42 was significant (*p* < 0.001; ***), GMR> Aβ42 and GMR>Aβ42+*hpo* (gain-of-function) was significant (*p* < 0.001; ***), and between GMR> Aβ42 and GMR>Aβ42+*hpo*^*RNAi*^ (loss-of-function) was significant (*p* < 0.001; ***). The *p*-value obtained from Student's *t*-test between GMR>Aβ42 and GMR> Aβ42+*wts* was significant (*p* < 0.05; *), between GMR> Aβ42 and GMR> Aβ42+*wts*^*RNAi*^ was significant (*p* < 0.001; ***), between GMR> Aβ42 and GMR> Aβ42+*yki* was significant (*p* < 0.001; ***), and between GMR> Aβ42 and GMR> Aβ42+*yki*^*RNAi*^ was significant (*p* < 0.001; ***). **(B–S)** shows the extent of cell death based on TUNEL assays (red, gray) in indicated genotypes in imaginal discs, pupal retinae, and adult eyes. The eye discs **(B,E,H,K,N,Q)** were assessed for dying cells using TUNEL assay (red) and stained with Dlg (green) and embryonic lethal abnormal vision (ELAV; blue). The pupal retinae **(C,F,I,L,O,R)** assessed for dying cells using TUNEL assays (gray). Adult eye phenotypes are shown in panels **(D,G,J,M,P,S)**. Panels show the extent of cell death in **(B–D)** GMR> Aβ42+*hpo*, **(E–G)** GMR> Aβ42+*hpo RNAi*, **(H–J)** GMR> Aβ42 +*wts*, **(K–M)** GMR> Aβ42 +*wts*^RNAi^, **(N–P)** GMR> Aβ42 + *yki*^RNAi^, and **(Q–S)** GMR> Aβ42 + *yki*. The orientation of all imaginal discs is identical with posterior to the left and dorsal up. Magnification of all eye imaginal discs is 20×.

We tested if activation of Hippo signaling is responsible for triggering cell death of neurons in GMR> Aβ42 background. So we tested if other components of the pathway that act downstream of Hpo affect Aβ42-mediated neurodegeneration. We employed TUNEL staining that labels the fragmented DNA and thereby mark the nuclei of the dying neurons (White et al., 1994; Cutler et al., 2015). Misexpression of Aβ42 results in induction of cell death as evident from TUNEL-positive nuclei in the eye imaginal disc and the adult eye (Figure 4A). Activation of Hippo signaling by misexpressing *hpo* along with Aβ42 (GMR> Aβ42+ *hpo*) results in 2-fold increase in cell death (Figure 4A) as evident from the number of TUNEL-positive nuclei in eye imaginal disc (Figure 4B) and pupal retina (Figure 4C). The adult fly of GMR> Aβ42+*hpo* genotype failed to hatch out and exhibits a “no-eye” phenotype (Figure 4D). Similar effects were observed when the Hippo pathway was activated by overexpression of *wts* (GMR> Aβ42+*wts*^13F^; Figures 4A,H–J) or downregulation of *yki* (GMR> Aβ42+*yki*^RNAi^; Figures 4A,N–P). In contrast, downregulation of Hippo signaling by *hpo*^RNAi^ (GMR> Aβ42+*hpo*^RNAi^; Figures 4A,E–G) and *wts*^RNAi^ (GMR> Aβ42+*wts*^RNAi^; Figures 4A,K–M) or overexpression of *yki* (GMR> Aβ42+*yki*; Figures 4A,Q–S) results in the converse phenotype of a significant rescue as evident from the highly reduced cell death (Figure 4A) in the eye imaginal disc (Figures 4A,E,K,Q), pupal retina (Figures 4F,L,R), and in the adult eye (Figures 4G,M,S), respectively. These data suggest that other pathway components, specially the effector Yki, also modify the effects of Aβ42-mediated neurodegeneration. Genetic interactions also suggest that the Hippo pathway acts downstream of Aβ42 accumulation. Aβ42-mediated neurodegeneration is dependent on the JNK signaling pathway activity (Tare et al., 2011). Therefore, we explored the JNK pathway as we have previously shown a similar downstream role of JNK signaling in Aβ42-mediated cell death (Tare et al., 2011).

### JNK Activity Is Modulated by Hippo Pathway Levels in Amyloid-Beta 42-Mediated Neurodegeneration

**Figure 5:**
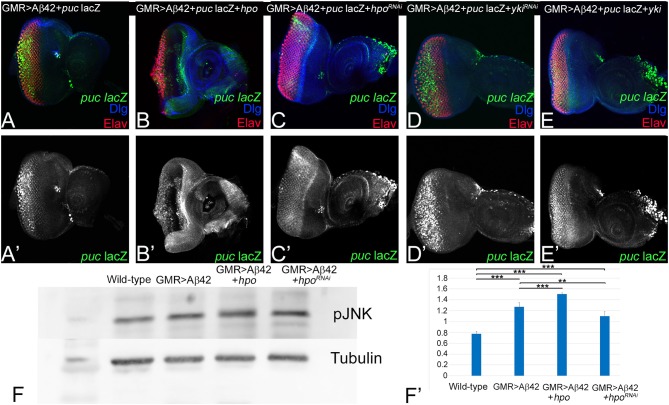
Activation of Hippo signaling upon amyloid-beta 42 (Aβ42) accumulation also activates c-Jun-amino-terminal kinase (JNK) signaling. **(A–E)** shows the expression of embryonic lethal abnormal vision (ELAV; red), JNK signaling pathway reporter *puc*-lacZ (green, gray), and discs large (Dlg; blue) in eye discs from **(A,A')** glass multiple repeat (GMR)> Aβ42, **(B,B')** GMR> Aβ42 + *hpo*, **(C,C')** GMR> Aβ42 + *hpo*^RNAi^, **(D,D')** GMR> Aβ42 + *yki*^RNAi^, and **(E,E')** GMR> Aβ42 + *yki*. For comparison between genotypes, **(A'–E')** shows *puc-lacZ* (Gray) levels. **(F)** A semiquantitative Western blot is presented to show phospho-JNK (p-JNK) levels in the wild-type, GMR> Aβ42, GMR> Aβ42 + *hpo*, and GMR> Aβ42 + *hpo*^RNAi^ background. The samples were loaded in the following sequence: Lane 1-Molecular weight marker, Lane 2-Wild-type (Canton-S), Lane 3-GMR> Aβ42, Lane 4-GMR> Aβ42+*hpo* (gain-of-function), Lane 5-GMR> Aβ42+*hpo*^*RNAi*^ (loss-of-function). Alpha-tubulin is used as a loading control, and **(F')** graph shows the quantification of p-JNK levels, which were calculated from a set of three (*n* = 3) in wild-type and other indicated genotypes from the Western blot **(F)**. The *p*-values for estimation of p-JNK levels in all combination in a semiquantitative Western blot was calculated in a set of three (*n* = 3) using Student's *t*-test in Microsoft Excel software. The *p*-value between wild-type and GMR> Aβ42 was significant (*p* < 0.001; ***), wild-type and GMR> Aβ42+*hpo* was significant (*p* < 0.001; ***), and between wild-type (Canton-S) and GMR> Aβ42+*hpo*^*RNAi*^ (loss-of-function) was significant (*p* <0.001; ***). The *p*-value between GMR> Aβ42 and GMR> Aβ42+*hpo* was significant (*p* < 0.001; ***) and between GMR> Aβ42 and GMR> Aβ42+*hpo*^*RNAi*^ was significant (*p* < 0.01; **).

Hippo signaling can activate JNK signaling (Ma et al., 2015, 2017). We first tested if changes in Hippo signaling activity affect JNK signaling activity in the GMR> Aβ42 background. *puc*-lacZ serves as a reporter for the JNK signaling activity (Martin-Blanco et al., 1998). Earlier we reported that *puc-*lacZ is robustly induced in Aβ42 (GMR> Aβ42) background, suggesting increased JNK activity (Figures 5A,A′; Tare et al., 2011). As compared to GMR> Aβ42, gain-of-function of *hpo* (GMR> Aβ42+ *hpo, puc*-lacZ) results in a strong upregulation of *puc*-lacZ reporter expression (Figures 5B,B′), whereas downregulation of *hpo* by misexpression of *hpo*^RNAi^ (GMR> Aβ42+ *hpo*^RNAi^, *puc-lacZ*) results in downregulation of *puc*-lacZ reporter expression (Figures 5C,C′). *puc*-lacZ expression coincides with the area of highest neuronal loss. To study the effects of downstream pathway components, we checked effects of upregulation or downregulation of Yki. Blocking Hippo signaling by *yki* misexpression (GMR> Aβ42+ *yki, puc-lacZ*) shows robust induction of *puc*-lacZ reporter in GMR expression domain (Figures 5 E,E′). Consistent with this, downregulation of Yki (GMR> Aβ42+ *yki*^*RNAi*^, *puc-lacZ*) did not induce high levels of *puc-lacZ* (Figures 5 D,D′). Our data suggest that activation of Hippo signaling in GMR> Aβ42 background results in enhancement of neurodegeneration, which is accompanied by induction of JNK signaling pathway.

We further verified our immunohistochemistry results with a semiquantitative Western blot to assess levels of phospho-JNK, the activated form of JNK (Mehan et al., 2011). pJNK levels were compared in protein extracts made from eye discs from wild type, GMR> Aβ42, GMR> Aβ42+ *hpo*, and GMR> Aβ42+ *hpo*^RNAi^. In comparison to the wild type, p-JNK levels were upregulated in GMR> Aβ42, GMR> Aβ42+ *hpo* background, whereas it was reduced in GMR> Aβ42+ *hpo*^RNAi^ background. The alpha-tubulin served as the loading control. The quantification of pJNK levels shows that compared to GMR> Aβ42, pJNK levels are higher when Hippo pathway is activated (GMR> Aβ42+ *hpo*; Figures 5F,F′). Taken together, these data present evidence for activation of both Hippo and JNK pathways during Aβ42-mediated neurodegeneration. Genetic interaction and Western blot analysis shows that Hippo pathway can activate JNK signaling in the GMR> Aβ42. However, given the complex context-dependent nature of interactions between the Hippo and JNK pathways, it is important to test whether JNK signaling pathway can also affect Hippo signaling pathway.

### Activation of Hippo Signaling in Amyloid-Beta 42 Background Is Dependent on c-Jun-Amino-Terminal Kinase Signaling Pathway

**Figure 6:**
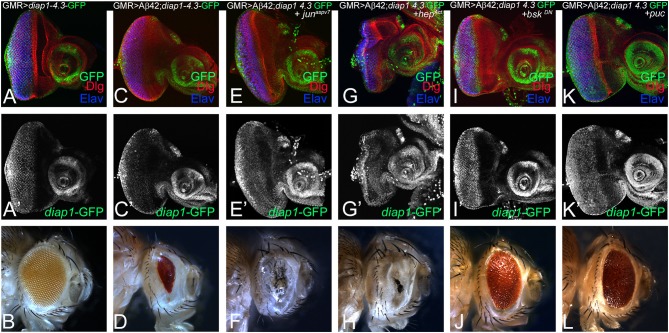
Activation of c-Jun-amino-terminal kinase (JNK) signaling pathway in amyloid-beta 42 (Aβ42) background activates Hippo pathway. Eye imaginal discs from larvae of indicated genotypes assessed for changes in expression of *diap1-4.3*-green fluorescent protein (GFP; green), a reporter for Hippo pathway, are shown. All discs show expression of discs large (Dlg; red), *diap1*-4.3-GFP (green), and embryonic lethal abnormal vision (ELAV; blue). **(A,A')** show wild-type control glass multiple repeat (GMR)> *diap1*-4.3-GFP eyes discs and **(B)** wild-type adult eye phenotypes; and **(C,C')** GMR> Aβ42 eye discs and **(D)** adult eye phenotype. **(E–K)** shows effects of modulating JNK activity on Hippo pathway reporter *diap1-4.3*-GFP in eye-antennal imaginal disc of **(E,E')** GMR> Aβ42 + *jun*^aspv7^, **(G,G')** GMR> Aβ42 + *hep*^Act^, **(I,I')** GMR> Aβ42 + *bsk*^DN^, **(K,K')** GMR> Aβ42 + *puc* backgrounds. Note that **(E,E',G,G')** *diap1-4.3*-GFP exhibits robust induction when JNK signaling is activated, whereas **(I,I',K,K')** *diap1-4.3*-GFP levels are significantly reduced when JNK signaling is downregulated in GMR> Aβ42 background. The adult eye phenotypes associated with **(D)** GMR> Aβ42, **(F)** GMR> Aβ42+ *jun*^aspv7^, **(H)** GMR> Aβ42+ *hep*^Act^, **(J)** GMR> Aβ42+ *bsk*^DN^, and **(L)** GMR> Aβ42+ *puc*. Note that **(J,L)** downregulation of JNK signaling showed significant rescues in the adult eye phenotypes, whereas **(F,H)** activation of JNK signaling enhanced the neurodegenerative phenotype of **(D)** GMR> Aβ42.

To further explore the relationship between Hippo and JNK signaling pathways in Aβ42-mediated neurodegeneration, we tested Hippo signaling activity when levels of JNK signaling pathway are modulated in GMR> Aβ42 background. *diap1*-4.3-GFP serves as a functional readout of the Hippo signaling pathway (Ren et al., 2010). In comparison to *diap1*-4.3-GFP expression in the control eye disc (Figures 6A,A′), the *diap-1*-4.3-GFP reporter is downregulated in GMR> Aβ42 background (Figures 6C,C′). The GMR> Aβ42 adults exhibit strong neurodegeneration phenotype in the eye (Figure 6D) as compared to the wild-type adult eye (Figure 6B). Upregulation of JNK signaling activity by misexpressing activated Jun in the GMR> Aβ42 background (GMR> Aβ42+ *jun*^aspv7^; Figures 6E,E′) or by expression of activated Hep (GMR> Aβ42+ *hep*^Act^; Figures 6G,G′) exhibits downregulation of *diap1*-4.3-GFP reporter activity. However, when JNK signaling is downregulated by misexpression of dominant-negative Bsk in GMR> Aβ42 background (GMR> Aβ42+ *bsk*^DN^; Figures 6I,I′) or Puc (GMR> Aβ42+ *puc*; Figures 5K,K′), it results in a strong upregulation of *diap1*-4.3-GFP reporter expression in the GMR domain. This correlates with the adult eye phenotypes where activating JNK signaling by misexpressing activated Jun (GMR> Aβ42+ *jun*^aspv7^; Figure 5F) and Hep (GMR> Aβ42+ *hep*^Act^; Figure 6H) enhances the Aβ42-mediated neurodegeneration, whereas downregulating JNK signaling by misexpressing dominant negative Bsk (GMR> Aβ42+ *bsk*^DN^; Figure 6J) and *puc* (GMR> Aβ42+ *puc*; Figure 6L) rescues the Aβ42-mediated neurodegeneration in adult eye. Thus, modulating JNK signaling can (i) modulate neurodegeneration phenotype of GMR> Aβ42 and (ii) also regulate Hippo signaling as evident from the changes in *diap1*-4.3-GFP reporter expression in the eye. Our data suggest that both Hippo and JNK can affect each other in Aβ42-mediated neurodegeneration.

### Hippo and c-Jun-Amino-Terminal Kinase Signaling May Interact in Amyloid-Beta 42-Mediated Neurodegeneration

**Figure 7:**
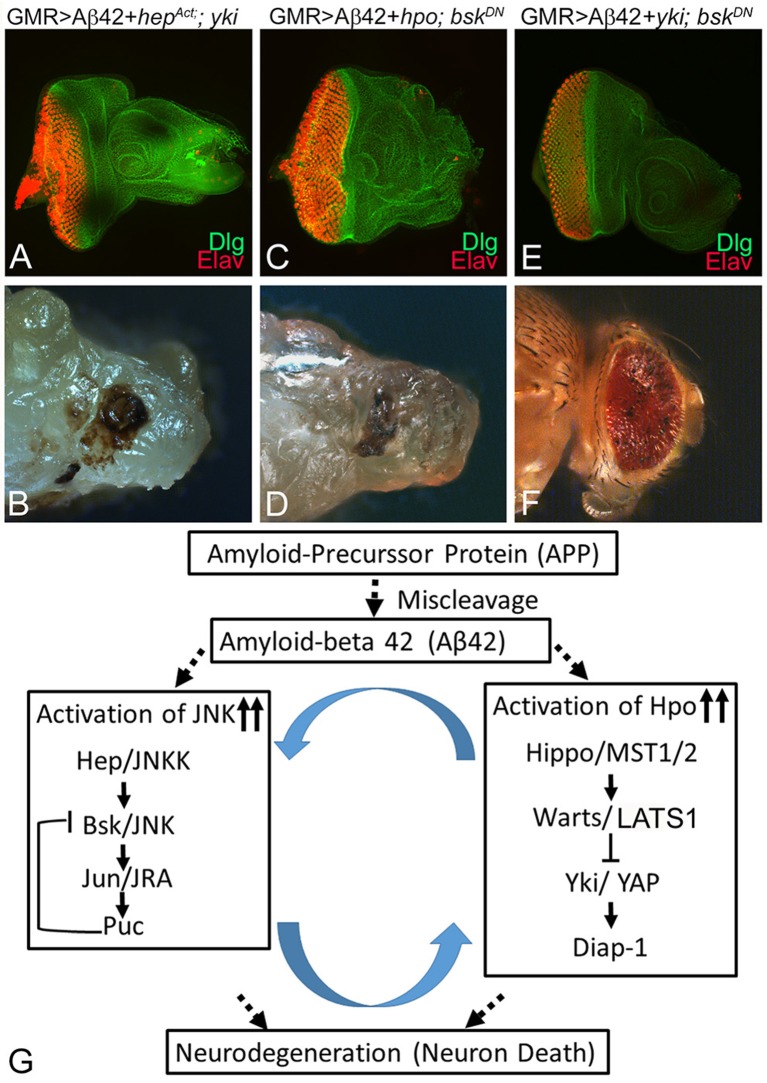
Hippo and c-Jun-amino-terminal kinase (JNK) signaling act synergistically to induce amyloid-beta 42 (Aβ42)-mediated neurodegeneration. Panels show eye discs stained with discs large (Dlg; green) and embryonic lethal abnormal vision (ELAV; red) and the resulting adult eye phenotypes when JNK or Hippo pathway is blocked. **(A,B)** Activation of JNK signaling along with inactivation of Hippo signaling in the glass multiple repeat (GMR)> Aβ42 background (GMR> Aβ42+*hep*^Act^+*yki*) does not rescue the neurodegenerative phenotype. **(C,D)** Activation of Hippo signaling along with inactivation of JNK signaling in the GMR> Aβ42 background (GMR> Aβ42+*hpo*+*Bsk*^DN^) does not rescue the neurodegenerative phenotype. **(E,F)** Inactivation of both JNK signaling and Hippo signaling (GMR> Aβ42+ *yki* + *Bsk*^*DN*^) together in the GMR> Aβ42 background exhibits significant rescue of the neurodegenerative phenotype. **(G)** A model that reconciles the data from this study, which shows that Hippo signaling and JNK signaling act synergistically *via* a positive feedback loop to induce Aβ42-mediated neurodegeneration.

To explore the relationship between Hippo and JNK pathways further, we used genetic epistasis. We sampled the effects at two developmental stages of third instar eye-antennal imaginal disc and the adult eye. In an Aβ42 background (GMR> Aβ42), we activated JNK signaling and blocked Hpo signaling at the same time by misexpression of activated *hep* and *yki* (GMR> Aβ42+*he*p^Act^ +*yki*). This resulted in stronger neurodegeneration in the eye imaginal disc (Figure 7A) and the adult eye (Figure 7B) as compared to GMR> Aβ42 alone. These flies failed to hatch out of the pupal case and exhibited a strong neurodegenerative phenotype. We also tested the effects of activation of Hippo signaling and downregulation of JNK signaling in the GMR> Aβ42 background by misexpressing *hpo* and dominant negative *bsk* (GMR> Aβ42+*hpo*+*bsk*^DN^). This also resulted in strong neurodegeneration both in the eye imaginal disc (Figure 7C) as well as the adult eye (Figure 7D). These flies also failed to hatch out of the pupal case and were dissected out from their pupal case. These data suggest that increasing levels of either Hippo or JNK signaling pathways do not compensate for the downregulation of the other. Therefore, both pathways may act in a feed forward/feedback loop. We further tested this hypothesis by blocking both Hippo and JNK signaling pathways by misexpressing *yki* and *bsk*^*DN*^, which resulted in a rescue in the eye imaginal disc (Figure 7E) as well as the adult eye (Figure 7F). These adults hatch out from the pupal case, although they showed some black necrotic spots. Our data suggest that the Hippo and JNK pathways interact synergistically in a positive feedback loop during Aβ42-mediated neurodegeneration (Figure 7G).

## Discussion

AD, an age-related progressive neurodegenerative disorder, manifests by progressive neuronal loss, brain atrophy, and cognitive impairments (Barnes and Yaffe, 2011). However, the mechanism of neurodegeneration observed in AD and related dementia (ADRD) has not been fully understood (Sarkar et al., 2016; Deshpande et al., 2019). Accumulation of Aβ42 plaques over a period of time triggers neuronal death due to the induction of aberrant signaling in neurons (Hardy, 2009; Tare et al., 2011). In addition, the causal genetic defects in AD are not due to a single gene mutation but are thought to involve genetic alterations that cause impairment of several signaling pathways (Sarkar et al., 2016). Currently, identification and characterization of downstream target(s) of aberrant signaling induced by abnormally high levels of Aβ42 are a growing area of research.

Several animal AD models have shown promise; however, our *Drosophila* model allows us to test other signaling pathways using genetic epistasis approaches (Pandey and Nichols, 2011; Lenz et al., 2013; Sabbagh et al., 2013; Sarkar et al., 2016; Deshpande et al., 2019). We previously reported the neuroprotective effects of the apical basal polarity gene *crumbs* (*crb*) (Moran et al., 2013; Steffensmeier et al., 2013) and the homeotic gene *teashirt* (*tsh*) (Moran et al., 2013) in Aβ42-mediated neurodegeneration. Interestingly, these genetic modifiers are members of the Hippo signaling pathway. In the present study, we employed our *Drosophila* eye model of AD to identify genetic modifiers in a forward genetic screen using the molecularly defined deficiencies set. We identified a deficiency *Df(2R)BSC782/*+, which can rescue Aβ42-mediated neurodegeneration. This deficiency uncovers *hpo* and other genes (Figure 1), and we confirmed that *hpo* was the specific genetic modifier of Aβ42 phenotypes. These data strongly support a role of Hippo signaling in Aβ42-mediated neurodegeneration.

### Hippo Acts Downstream of Amyloid-Beta 42 Plaque

We analyzed accumulation of Aβ42 protein in differentiating retinal neurons of eye imaginal disc from various genetic backgrounds of GMR> Aβ42 alone as well as where Hippo levels have been modulated. The rescue of the neurodegeneration phenotype due to downregulation of Hippo signaling occurs by a mechanism downstream of Aβ42 accumulation (Figure 2). We tested the levels of *diap1-*4.3-GFP (Ren et al., 2010) and *ex*-lacZ (Boedigheimer et al., 1993; Hamaratoglu et al., 2006), which serve as the reporter for Hippo signaling, in GMR> Aβ42 background (Figure 3). When Hippo signaling is activated, it triggers cell death (Verghese et al., 2012), and *diap1*-4.3-GFP and *ex*-lacZ levels are downregulated. We found that *diap1*-4.3-GFP and *ex*-lacZ levels were downregulated in GMR> Aβ42 background, which suggests that Hippo signaling is activated (Figure 3). Furthermore, we found that cell death levels were increased in GMR> Aβ42 background when Hippo levels were upregulated and *vice versa* (Figure 4). Thus, our study suggests that Aβ42 plaques induce Hippo signaling to trigger neurodegeneration (Figure 7G).

### Activation of c-Jun-Amino-Terminal Kinase Signaling

Previously, we and others have shown that JNK signaling is activated in GMR> Aβ42 background. (Tare et al., 2011; Sarkar et al., 2016). It is known that Hippo can regulate JNK signaling (Ma et al., 2015, 2017). To understand if the two pathways work independently or together in triggering Aβ42-mediated neurodegeneration, we tested the reporters like *puc*-lacZ, a reporter for JNK activity in our study (Martin-Blanco et al., 1998). We found that JNK signaling is activated during Aβ42-mediated neurodegeneration. Furthermore, when we activate Hippo signaling in GMR> Aβ42 background, we found that the reporters for JNK signaling were robustly activated (Figure 5). We also tested the levels of p-JNK and found that when Hippo pathway is downregulated, JNK signaling is also reduced. Thus, it is possible that activation of Hippo signaling can trigger activation of JNK signaling in GMR> Aβ42 background.

Furthermore, we explored the converse relation. We tested if JNK signaling can activate Hippo signaling in GMR> Aβ42 background (Figure 6). Interestingly, we found that activation of JNK signaling in GMR> Aβ42 background further enhances the neurodegenerative phenotype of GMR> Aβ42. Interestingly, the reporter of Hippo signaling pathway, *diap1*-GFP, showed robust activation where we activated JNK signaling in GMR> Aβ42 background (Figure 6). This suggests that activation of JNK signaling can further enhance the effects of Hippo activation and *vice versa*. Also, it suggests that these two pathways can in turn activate each other (Figures 5, 6). We then explored the roles of Hippo and JNK signaling in Aβ42-mediated neurodegeneration to understand if these key pathways interact or act independently.

### Positive Feedback Loop of Hippo and c-Jun-Amino-Terminal Kinase Signaling Regulate Neuroprotective Function

We previously reported the neuroprotective effects of the apical basal polarity gene *crumbs* (*crb*) (Moran et al., 2013; Steffensmeier et al., 2013) and the homeotic gene *teashirt* (*tsh*) (Moran et al., 2013), both members of the Hippo signaling pathway. However, the neuroprotective function of Hippo or JNK signaling interactions together has not been fully understood. We tested if neuronal death observed in Aβ42-mediated neurodegeneration uses both JNK and Hippo signaling independently or in epistatic interactions to trigger neurodegeneration. We employed classical genetic approaches to determine this relation between two signaling pathways. We found that if we activate JNK signaling and downregulate Hippo signaling at the same time in GMR> Aβ42 background or *vice versa*, it does not rescue neurodegenerative phenotypes both in the third instar eye-antennal imaginal disc and the adult eye (Figures 7A–D). This observation fits with our prior results (Figures 5, 6) that activation of one pathway can activate the other. Finally, we found that blocking both Hippo signaling and the JNK signaling at the same time exhibits a significant rescue of the GMR> Aβ42-mediated neurodegeneration during both developmental stages (Figures 7E,F). Based on our results, we propose a model that accumulation of Aβ42 triggers Hippo and JNK signaling (Figure 7G). There is ample evidence for the involvement of JNK signaling in AD (Yarza et al., 2015). We report a role for Hippo signaling in Aβ42-mediated neurodegeneration. Furthermore, based on our genetic interaction studies, we found that JNK and Hippo signaling are involved in a positive feedback loop in Aβ42-mediated neurodegeneration, and inactivation of both cascades rescues the phenotype (Figure 7G). Both of these pathways are crucial for normal development and in disease pathology. It will be interesting to explore the therapeutic value of pathway components in the future.

### Novel Role of Hippo in Neuroprotection

A growing body of epidemiological evidence and molecular investigations has shown some interesting links between cancer and AD (Battaglia et al., 2019; Nudelman et al., 2019). For example, several studies have shown that autophagy, ubiquitin proteasome system, and cell death are common biological hallmarks shared by AD and cancer (Nudelman et al., 2019). Hippo signaling has been shown to regulate organ size growth, cell proliferation, and cell death (Justice et al., 1995; Xu et al., 1995; Tapon et al., 2002; Harvey et al., 2003; Jia et al., 2003; Pantalacci et al., 2003; Wu et al., 2003; Lai et al., 2005; Kango-Singh and Singh, 2009; Halder and Camargo, 2013; Snigdha et al., 2019) and neural development (Wittkorn et al., 2015). Recently, Hippo signaling was implicated in many disease models where it plays a role in apoptosis, autophagy, regeneration, and cell survival (Pfleger, 2017; Ma et al., 2019; Sahu and Mondal, 2019). Thus, it is interesting to find a role for Hippo pathway in Aβ42-mediated neurodegeneration. Since several components of the Hippo pathway are ubiquitously expressed in flies, it is possible that Hippo signaling downregulation not only promotes cell proliferation but also may be providing neuroprotection. In case of neurons, which are postmitotic cells, the cell survival or neuroprotective function is utilized. Activation of MST1/2 has been associated with the progression of AD, where amyloid precursor protein (APP) promotes the interaction of transcription factor FOXO3a with MST1, triggering Bim (a proapoptotic member of te Bcl-2 family)-mediated neuronal death (Sanphui and Biswas, 2013). Recently, it has been shown that the amyloid β precursor proteins (AβPPs) like APLP1 and APLP2 can use YAP/TAZ, the mammalian orthologs of Yki, as signal transducers (Orcholski et al., 2011). Improper cleavage of amyloid precursor proteins (APP) by beta- and gamma secretase produces amyloid-beta polypeptide(s), which are prone to aggregation and are involved in AD. Dysfunction of PP2ACα, a key member of the protein phosphatase family that negatively regulates Hippo pathway, also results in AD-like conditions (Liu et al., 2018). Hippo pathway is also implicated in other neurodegenerative disorders, for example, MST1/2, the mammalian orthologs of Hpo, play a key role in amyotrophic lateral sclerosis (ALS). Loss of MST1 (e.g., in MST1 knockout mice) shows increased motor neuron viability, delayed symptom onset, and extended survival (Lee et al., 2013). Other examples include Huntington disease (Mueller et al., 2018) and retinal degeneration (Murakami et al., 2014). Thus, our findings on the Hippo and JNK pathways open new avenues of research in the AD field and may help find better targets for devising therapeutic interventions in the future.

## Data Availability Statement

All datasets generated for this study are included in the article/supplementary material.

## Author Contributions

MI and MT performed the experiments and data analysis. AdS, OP, NG, MR, and PD performed the experiments. MK-S performed data analysis and manuscript writing. AS developed the concept, performed data analysis and manuscript writing.

### Conflict of Interest

The authors declare that the research was conducted in the absence of any commercial or financial relationships that could be construed as a potential conflict of interest.

## Abbreviations

AD: Alzheimer's disease
Aβ42: amyloid-beta 42
Hpo/MST1/2: Hippo
Yki/YAP: Yorkie
Wts/LATS1: Warts
Sav/SAV1/WW45: Salvador
Mobs/hMob1: Mob-as-tumor-suppressor
Sd/TEAD1: Scalloped
DIAP1/BIRC5: *Drosophila* inhibitor of apoptosis
*rpr*: *reaper*
*hid*: *head involution defective*
JNK: c-Jun-amino-terminal kinase
MAPK: mitogen-activated protein kinase
Egr/TNFα: Eiger
Hep/JNKK: *hemipterous*
Bsk/JNK: *basket*
dJun/c-Jun: Jun-related antigen
*puc*: *puckered*
GMR: glass multiple repeat
PBS: phosphate buffered saline
ELAV: embryonic lethal abnormal vision
Dlg: discs large.
